# Validation of age- and sex-dependent association of uric acid and incident hypertension in rural areas

**DOI:** 10.1186/s40885-022-00206-5

**Published:** 2022-09-01

**Authors:** In Jae Kim, Woohyeun Kim, Tae Hwa Go, Dae Ryong Kang, Jang-Young Kim, Eung Ju Kim

**Affiliations:** 1 Department of Cardiology, Namwon Medical Center, Namwon, Republic of Korea; 2 Division of Cardiology, Department of Internal Medicine, Hanyang University College of Medicine, 222-1, Wangsimni-ro, Seongdong-gu, 04763 Seoul, Republic of Korea; 3 Center of Biomedical Data Science, Yonsei University Wonju College of Medicine, Wonju, Republic of Korea; 4 Department of Cardiology, Yonsei University Wonju College of Medicine, Wonju, Republic of Korea; 5 Cardiovascular Center, Korea University Guro Hospital, Seoul, Republic of Korea

**Keywords:** Uric acid, Hyperuricemia, Hypertension

## Abstract

**Background:**

A previous study based on urban areas suggested the age- and sex-dependent association of uric acid (UA) and incident hypertension. We aimed to investigate whether this association is valid even in rural areas with different lifestyle.

**Methods:**

Data from the cardiovascular disease association study, a prospective cohort study based on rural residents, was analyzed. A total of 4,592 subjects (mean age, 60.1 ± 9.5 years; men, 37.7%) without hypertension were included. We first investigated whether UA was a risk factor for incident hypertension using Cox regression, and then compared the relative risk by stratification according to age and sex.

**Results:**

During the follow-up period (mean, 2.0 years), 579 subjects (12.6%) were newly diagnosed with hypertension. The risk factors for incident hypertension were age (Hazard ratios [HR] for ≥ 65, 1.26), systolic blood pressure (HR per 1 mmHg increase, 1.07), and serum UA concentration (HR per 1 mmHg increase, 1.10). The risk of UA-related incident hypertension was higher in the non-elderly than in the elderly for both men and women (HR, 1.74 for non-elderly men; 1.88 for non-elderly women; 1.66 for elderly men; 1.10 for elderly women). Even after adjusting for multiple confounders, the risk of UA-related incident hypertension was significantly higher in non-elderly women (HR, 1.59; *P* < 0.05).

**Conclusions:**

Age- and sex-dependent association of UA with incident hypertension suggested in cohort study based on urban areas was consistently found in rural areas as well. In particular, non-elderly women were at a higher risk for UA-related incident hypertension.

**Supplementary information:**

The online version contains supplementary material available at 10.1186/s40885-022-00206-5.

## Background

The link between uric acid (UA) and hypertension has long been of interest. Since it was first suggested more than a century ago that UA may play a role in the pathogenesis of hypertension [1], many studies on the association between hypertension and UA have been conducted. Nevertheless, the results on the association between hypertension and UA in many previous studies were not clearly consistent because there were many differences in age, sex, and various variables related to lifestyle that affect UA in each study.

Recent meta-analyses had reported that hyperuricemia and elevated UA are associated with the risk of hypertension [2, 3]. The authors suggested that this association may be relatively greater in younger age and in women. However, significant differences in age and sex composition among the study populations included in these analyses remained limited in concluding the role of age and sex in the risk of UA-associated hypertension. Recently, we reported an age- and sex- dependent association of UA and incident hypertension in a community-based prospective cohort study [4]. However, UA is affected by lifestyle [5–11], and in that study, there was a limitation that these confounders were not sufficiently corrected. Since it is impossible to completely adjust the lifestyle in a prospective study, we aimed to determine whether the results of our previous study based on urban areas are also valid in this study based on rural areas.

## Methods

### Study population and design

We analyzed data from the cardiovascular disease association study (CAVAS) cohort, which is part of the Korean Genome and Epidemiology Study (KoGES) cohort. KoGES is a large-scale, prospective cohort study initiated by the Korean government, establishing an epidemiological study platform with a health database, and investigating the environmental etiology and prognosis of chronic diseases in Koreans through long-term follow-up. The CAVAS study, one of the six prospective cohort studies constituting KoGES, is a rural community-based study targeting residents living in rural areas that are different from in urban areas. It recruited the subjects aged 40–70 years from rural areas since 2004. All participants were interviewed for the sociodemographic information and medical history. Laboratory tests were performed by trained medical staff. Blood pressure was measured twice using a mercury sphygmomanometer and recorded as the average of the two values. The first measurement was taken after 5 min of rest, and the second measurement was taken at least 1 min apart. If the difference between the two measurements exceeded 5 mmHg, blood pressure measurements were continued until two consecutive measurements were comparable. Details of the KoGES and CAVAS study have been described previously [12].

**Fig. 1:**
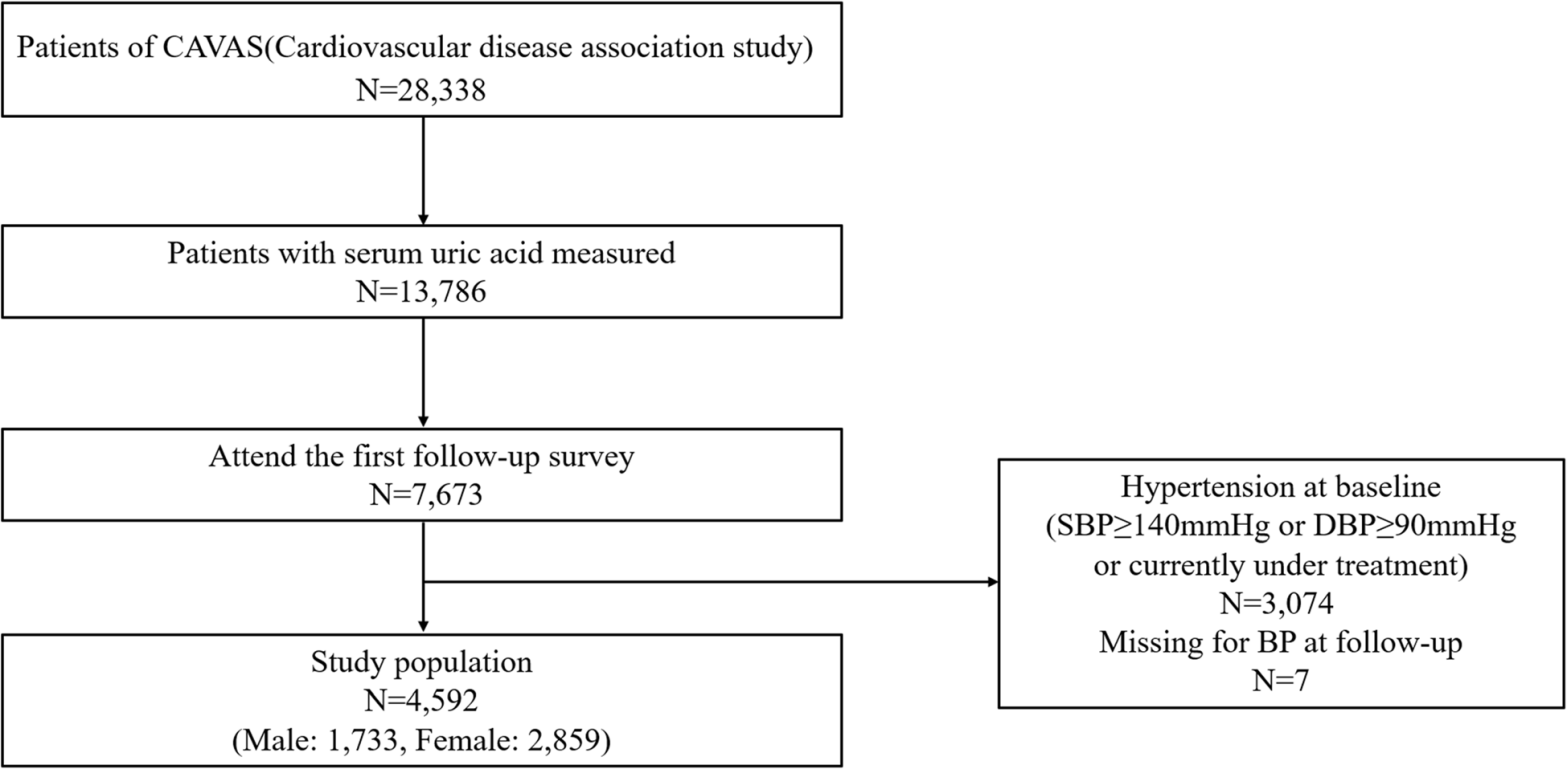
Flow chart of the study population. CAVAS, cardiovascular disease association study; SBP, systolic blood pressure; DBP, diastolic blood pressure; BP, blood pressure

Of the 28,338 subjects who participated in CAVAS, 7,673 subjects with UA measured participated in the first follow-up were included in this study. Finally, 4,592 subjects were analyzed, excluding those who had hypertension at baseline (*n* = 3,074) or those who did not have data on blood pressure (*n* = 7) (Fig. 1).

Hypertension (taking anti-hypertensives or having systolic blood pressure (BP) ≥ 140 mmHg or diastolic BP ≥ 90 mmHg), hyperuricemia (serum UA level > 7 mg/dL in men and > 6 mg/dL in women) [13, 14], smoking (smoked > 5 packs in his/her lifetime) [15, 16], and drinking (> 12 drinks in the past year) [17] were defined as in previous urban-based cohort study [4]. Incident hypertension was defined as the occurrence of hypertension during the follow-up period.

### Statistical analysis

We divided the entire study population into three groups according to the level of UA. Accordingly, tertile 1, 2, and 3 were defined as < 5.0 mg/dL, 5.0–6.0 mg/dL, and ≥ 6.0 mg/dL for men, and as < 3.8 mg/dL, 3.8–4.6 mg/dL, and ≥ 4.6 mg/dL for women, respectively. The risk of incident hypertension was analyzed using Cox regression for all subjects to determine whether UA is a risk factor for incident hypertension. Hazard ratios (HR) were adjusted for age, sex, baseline systolic and diastolic BP, diabetes, dyslipidemia, body mass index, estimated glomerular filtration rate, drinking/smoking status, and UA. To determine age- and sex-dependent association of UA and incident hypertension, the study population was stratified according to sex, age group, and UA level. All analyses were conducted using SAS ver. 9.4 (SAS Institute Inc., Cary, NC, USA). *P*-values < 0.05 were considered statistically significant.

## Results

### Baseline characteristics

Among the 4,592 subjects included in the analysis, 1,733 (37.7%) were male. The mean age was 60.1 ± 9.5 years, and mean follow-up duration was 2.0 years. Baseline characteristics are shown in Table 1. The mean concentration of UA was 4.8 ± 1.3 mg/dL, and 418 subjects (9.1%) had hyperuricemia. Subjects in tertile 3 tended to have higher BP, body mass index, low-density lipoprotein cholesterol, total cholesterol, and triglycerides, but lower estimated glomerular filtration rate and high-density lipoprotein cholesterol. They tended to be more drinkers.

**Table 1:** Baseline characteristics according to serum uric acid level

Variable	Total (*n* = 4,592)	Tertile 1 (*n* = 1,436)	Tertile 2 (*n *= 1,548)	Tertile 3 (*n* = 1,608)	*P*-value^a)^
Male sex	1,733 (37.7)	564 (39.3)	550 (35.5)	619 (38.5)	0.08
Age (yr)	60.1 ± 9.5	60.6 ± 9.6	59.3 ± 9.5	60.3 ± 9.4	< 0.05
40–64	2,926 (63.7)	864 (60.2)	1,044 (67.4)	1,018 (63.3)	
> 65	1,666 (36.3)	572 (39.8)	504 (32.6)	590 (36.7)	
Smoking status					< 0.05
Non-smoker	2,401 (67.5)	771 (67.1)	844 (70.6)	786 (65.0)	
Ex-smoker	578 (16.3)	182 (15.8)	193 (16.1)	203 (16.8)	
Current smoker	576 (16.2)	197 (17.1)	159 (13.3)	220 (18.2)	
Drinking status					< 0.05
Non-drinker	2,409 (52.5)	798 (55.6)	830 (53.6)	781 (48.6)	
Ex-drinker	294 (6.4)	90 (6.3)	90 (5.8)	114 (7.1)	
Current drinker	1,886 (41.1)	547 (38.1)	628 (40.6)	711 (44.3)	
Height (cm)	1,57.6 ± 8.3	157.3 ± 8.4	157.5 ± 8.3	157.9 ± 8.3	0.15
Weight (kg)	59.7 ± 9.7	57.7 ± 9.2	59.4 ± 9.5	61.7 ± 10.0	< 0.05
Body mass index (kg/m^2^)	24.0 ± 3.0	23.3 ± 3.0	23.9 ± 2.9	24.7 ± 3.0	< 0.05
Systolic blood pressure (mmHg)	116.5 ± 11.6	116.0 ± 11.8	116.0 ± 11.5	117.4 ± 11.4	< 0.05
Diastolic blood pressure (mmHg)	74.6 ± 7.9	73.9 ± 8.1	74.6 ± 8.0	75.1 ± 7.6	< 0.05
Fasting blood glucose (mg/dL)	98.9 ± 23.5	100.6 ± 31.1	97.7 ± 19.9	98.5 ± 18.1	< 0.05
eGFR (mL/min/1.73 m^2^)	76.4 ± 11.3	79.4 ± 11.2	77.1 ± 10.3	73.0 ± 11.5	< 0.05
Total cholesterol (mg/dL)	197.4 ± 35.7	193.2 ± 35.1	196.0 ± 34.8	202.5 ± 36.4	< 0.05
High-density lipoprotein (mg/dL)	45.3 ± 10.8	46.6 ± 10.8	45.6 ± 10.9	43.9 ± 10.6	< 0.05
Low density lipoprotein (mg/dL)	124.0 ± 32.9	121.8 ± 31.6	123.4 ± 31.7	126.5 ± 34.9	< 0.05
Triglyceride (mg/dL)	140.5 ± 87.2	124.0 ± 69.5	134.8 ± 87.2	160.6 ± 97.0	< 0.05
Diabetes mellitus	438 (9.5)	161 (11.2)	123 (8.0)	154 (9.6)	< 0.05
Dyslipidemia	2,988 (65.1)	835 (58.2)	958 (61.9)	1,195 (74.3)	< 0.05
Serum uric acid (mg/dL)	4.8 ± 1.3	3.6 ± 0.7	4.6 ± 0.7	6.0 ± 1.2	< 0.05
Hyperuricemia	418 (9.1)			418 (26.0)	< 0.05

Data are presented as number (%) or mean ± standard deviation

*eGFR* estimated glomerular filtration rate

^a)^Post-hoc analysis using Bonferroni method

### UA as a risk factor of hypertension

During the follow-up period, hypertension occurred in 579 of 4,592 subjects. The risk factors for incident hypertension were age, systolic BP, and serum UA concentration (Table 2). The risk of incident hypertension was 1.26 times higher in the elderly group over 65 years compared to the non-elderly group under 65 years old. The risk increased 1.07-fold for each 1 mmHg increase in systolic BP and 1.10-fold for each 1 mg/dL increase in UA.

**Table 2:** Risk factors for incident hypertension

Variable	Crude		Adjusted^a)^	
	Hazard ratio (95% CI)	*P*-value	Hazard ratio (95% CI)	*P*-value
Sex (vs. male)				
Female	1.00 (0.84–1.18)	0.97	1.23 (0.81–2.28)	0.41
Age (vs. 40–64)				
Over 65 years old	1.59 (1.35–1.87)	< 0.05	1.26 (1.02–1.56)	< 0.05
Smoking status (vs. non-smoker)				
Ex-smoker	1.17 (0.91–1.50)	0.22	1.21 (0.84–1.73)	0.30
Current smoker	0.96 (0.74–1.26)	0.79	1.19 (0.83–1.71)	0.33
Drinking status (vs. non-drinker)				
Ex-drinker	1.00 (0.71–1.41)	0.99	0.83 (0.53–1.30)	0.41
Current drinker	1.01 (0.85–1.19)	0.93	0.98 (0.79–1.22)	0.85
Body mass index (kg/m^2^)	1.05 (1.03–1.08)	< 0.05	1.01 (0.98–1.05)	0.48
Systolic blood pressure (mmHg)	1.07 (1.06–1.08)	< 0.05	1.07 (1.05–1.08)	< 0.05
Diastolic blood pressure (mmHg)	1.05 (1.04–1.06)	< 0.05	1.01 (0.99–1.02)	0.43
Diabetes mellitus	1.37 (1.07–1.75)	< 0.05	1.10 (0.83–1.47)	0.51
Dyslipidemia	1.29 (1.08–1.54)	< 0.05	1.06 (0.85–1.32)	0.60
eGFR (mL/min/1.73 m^2^)	1.79 (1.40–2.28)	< 0.05	1.24 (0.92–1.67)	0.16
Serum uric acid (mg/dL)	1.13 (1.06–1.19)	< 0.05	1.10 (1.02–1.20)	< 0.05

*CI* confidence interval, *eGFR* estimated glomerular filtration rate

^a)^Hazard ratios were estimated with adjustments for age, sex, baseline systolic and diastolic blood pressures, diabetes, dyslipidemia, body mass index, estimated glomerular filtration rate, drinking status, smoking status, and serum uric acid level

### Serum UA level and the risk of incident hypertension according to sex and age

Across the study populations, the risk of incident hypertension was highest in tertile 3, the group with the highest level of UA, for both men and women (HR, 1.74 for non-elderly men; 1.88 for non-elderly women; 1.66 for elderly men; 1.10 for elderly women) (Table 3). This was more pronounced in non-elderly women. After adjusting for multiple confounders, the risk of incident hypertension was significantly higher only in non-elderly women (HR, 1.59; 95% confidence interval, 1.01–2.50).

**Table 3:** Relative risk of hypertension according to uric acid level by sex and age

Group	UA	40-64 years					Over 65 years				
		Incidence case	Crude		Adjusted^a)^		Incidence case	Crude		Adjusted^a)^	
			HR (95% CI)	*P*-value	HR (95% CI)	*P*-value		HR (95% CI)	*P*-value	HR (95% CI)	*P*-value
Male	T1	23 (8.0)	1.00 (Reference)		1.00 (Reference)		32 (11.6)	1.00 (Reference)		1.00 (Reference)	
	T2	30 (9.4)	1.17 (0.68–2.02)	0.56	0.95 (0.49–1.85)	0.88	34 (14.7)	1.27 (0.79–2.06)	0.33	1.14 (0.63–2.07)	0.66
	T3	50 (13.9)	1.74 (1.06–2.85)	<0.05	1.74 (0.92–3.28)	0.09	50 (19.2)	1.66 (1.07–2.59)	0.02	1.51 (0.87–2.63)	0.14
Female	T1	41 (7.1)	1.00 (Reference)		1.00 (Reference)		52 (17.6)	1.00 (Reference)		1.00 (Reference)	
	T2	72 (9.9)	1.40 (0.95–2.05)	<0.05	1.22 (0.78–1.93)	0.38	43 (15.8)	0.89 (0.60–1.34)	0.59	0.91 (0.59–1.42)	0.68
	T3	88 (13.4)	1.88 (1.30–2.72)	<0.05	1.59 (1.01–2.50)	<0.05	64 (19.4)	1.10 (0.76–1.59)	0.61	0.94 (0.60–1.46)	0.77

*UA* uric acid, *HR* hazard ratio, *CI* confidence interval

Tertiles 1, 2, and 3, respectively, were defined as < 5.0, 5.0–6.0, and ≥ 6.0 mg/dL for men, and as < 3.8, 3.8–4.6, and ≥ 4.6 mg/dL for women

Incidences are presented as frequency (percentage)

^a)^adjusted for age, baseline systolic and diastolic blood pressure, diabetes, dyslipidemia, body mass index, estimated glomerular filtration rate, drinking status, and smoking status

## Discussion

The results of our study based on rural areas can be summarized as follows. In rural areas, an increase in UA was associated with incident hypertension. The risk of UA-related incident hypertension was significantly higher in non-elderly women. These results of this study were consistent with those of previous urban-based cohort study [4].

In previous meta-analyses, it has been reported that UA is associated with the risk of incident hypertension, regardless of traditional risk factors [2, 3]. However, in these analyses, the authors acknowledged that the statistical power to explain this relationship was weak in the elderly and that the statistical method, degree of adjustment, and residual confounding factors, which differed from study to study, were the limitations of the studies.

The weak statistical association in the elderly could be explained by a previous large cohort study based on urban areas [4], in which the association between UA and incident hypertension was age-dependent. In that study, the relative risk of incident hypertension according to UA levels differed between age groups, with the lower the age group, the higher the risk. In addition, they found that not only age but also sex play an important role in this association. They concluded that this association was age- and sex-dependent and strongest in younger women. However, in that study, as in previous studies [2, 3], residual confounders such as lifestyle remain a limitation.

UA is well known to be greatly affected by lifestyle [5–11]. However, since lifestyle such as dietary or behavioral patterns is difficult to objectively assess as well as difficult to categorize or quantify as a variable, it is impossible to completely adjust them in statistical analysis. Accordingly, we tried to determine whether age- and sex-dependent association between UA and incident hypertension is consistently observed in two different cohort groups, which are presumed to have different lifestyles. Because the lifestyles of urban and rural residents are inevitably different, the same analysis as in the previous urban-based cohort study was performed on CANVAS, a sub-study of KoGES targeting rural residents.

Although these two distinct cohorts cannot be directly compared, a numerical comparison of the study populations included in each study shows that the rural cohort had a higher mean age and more men, and higher rates of diabetes and hyperlipidemia and hyperuricemia, compared with the urban-based cohort (Supplementary Table 1). Despite these different baseline characteristics, the risk of UA-related incident hypertension was consistently high in both cohort studies, at least in non-elderly women.

Although the role of UA might be relatively high given the low prevalence of comorbidities such as chronic renal insufficiency and diabetes in non-elderly women, it is unclear why the risk of UA-associated hypertension is more pronounced in this group. Intriguingly, serum UA was more associated with metabolic syndrome [18], coronary heart disease [19], and renal insufficiency [20] in women than in men. Given these findings, it is conceivable that women tend to be more susceptible to UA-related cardiovascular disease.

Since most studies on the relationship between UA and the risk of incident hypertension have been conducted on relatively younger employed adults with an average age of 30–40 years, mainly men, few studies have focused on age and sex regarding this association [21–26]. As far as we know, one cross-sectional study reported that age and sex might be involved in the association between UA and hypertension [27], and one large-scale longitudinal study suggested that the risk of UA -related incident hypertension was age- and gender-dependent [4]. This study has clinical significance in confirming whether this association is still valid in populations with different characteristics.

This study also has some limitations. As with urban-based cohort study, age groups limited to 40–70 years and residual confounders undermine the value of this study. Also, considering that the concentration of UA can be affected and changed by various environmental factors, the significance of the value at the single point is inevitably limited. Unlike the urban-based cohort, the relatively small number of sample sizes and shorter follow-up duration made it impossible to analyze at a same level to the previous study, so direct comparison between urban and rural areas was not possible.

## Conclusions

Even in rural areas, non-elderly women were at a higher risk for UA-related incident hypertension. Age- and sex-dependent association of UA and incident hypertension is also valid in rural residents.

## Supplementary information

**Additional file 1: Supplementary Table 1.** Comparison of baseline characteristics between urban-based and rural-based cohorts.

## Data Availability

The datasets during and/or analyzed during the current study available from the corresponding author on reasonable request.

## Abbreviations

BP: Blood pressure
CAVAS: Cardiovascular disease association study
HR: Hazard ratios
KoGES: Korean Genome and Epidemiology Study
UA: Uric acid

## References

[1] Feig DI (2012). Hyperuricemia and hypertension. Adv Chronic Kidney Dis.

[2] Wang J, Qin T, Chen J, Li Y, Wang L, Huang H (2014). Hyperuricemia and risk of incident hypertension: a systematic review and meta-analysis of observational studies. PLoS ONE.

[3] Grayson PC, Kim SY, LaValley M, Choi HK (2011). Hyperuricemia and incident hypertension: a systematic review and meta-analysis. Arthritis Care Res (Hoboken).

[4] Kim W, Go TH, Kang DO, Lee J, Choi JY, Roh SY. N et al. Age and sex dependent association of uric acid and incident hypertension. Nutr Metab Cardiovasc Dis. 2021;31:1200–8. 10.1016/j.numecd.2020.12.015 33618926

[5] Gao X, Qi L, Qiao N, Choi HK, Curhan G, Tucker KL (2007). Intake of added sugar and sugar-sweetened drink and serum uric acid concentration in US men and women. Hypertension.

[6] Caliceti C, Calabria D, Roda A, Cicero AF (2017). Fructose intake, serum uric acid, and cardiometabolic disorders: a critical review. Nutrients.

[7] Yu S, Yang H, Guo X, Zhang X, Zhou Y, Ou Q (2016). Prevalence of hyperuricemia and its correlates in rural Northeast Chinese population: from lifestyle risk factors to metabolic comorbidities. Clin Rheumatol.

[8] Yang J, Liu Z, Zhang C, Zhao Y, Sun S, Wang S (2013). The prevalence of hyperuricemia and its correlates in an inland Chinese adult population, urban and rural of Jinan. Rheumatol Int.

[9] Xiong Z, Zhu C, Qian X, Zhu J, Wu Z, Chen L (2013). Serum uric acid is associated with dietary and lifestyle factors in elderly women in suburban Guangzhou in Guangdong province of south China. J Nutr Health Aging.

[10] Liu L, Lou S, Xu K, Meng Z, Zhang Q, Song K (2013). Relationship between lifestyle choices and hyperuricemia in Chinese men and women. Clin Rheumatol.

[11] Miao Z, Li C, Chen Y, Zhao S, Wang Y, Wang Z (2008). Dietary and lifestyle changes associated with high prevalence of hyperuricemia and gout in the Shandong coastal cities of Eastern China. J Rheumatol.

[12] Kim Y, Han BG, KoGES group (2017). Cohort profile: the Korean genome and epidemiology study (KoGES) consortium. Int J Epidemiol.

[13] Bardin T, Richette P (2014). Definition of hyperuricemia and gouty conditions. Curr Opin Rheumatol.

[14] Lin KC, Lin HY, Chou P (2000). Community based epidemiological study on hyperuricemia and gout in Kin-Hu, Kinmen. J Rheumatol.

[15] Jamal A, Phillips E, Gentzke AS, Homa DM, Babb SD, King BA (2018). Current cigarette smoking among adults: United States, 2016. MMWR Morb Mortal Wkly Rep.

[16] Yoon SS, Dillon CF, Illoh K, Carroll M (2016). Trends in the prevalence of coronary heart disease in the U.S.: National Health and Nutrition Examination Survey, 2001–2012. Am J Prev Med.

[17] Hartz SM, Oehlert M, Horton AC, Grucza RA, Fisher SL, Culverhouse RC (2018). Daily drinking is associated with increased mortality. Alcohol Clin Exp Res.

[18] Cicero AF, Fogacci F, Giovannini M, Grandi E, Rosticci M, D’Addato S (2018). Serum uric acid predicts incident metabolic syndrome in the elderly in an analysis of the Brisighella Heart Study. Sci Rep.

[19] Kim SY, Guevara JP, Kim KM, Choi HK, Heitjan DF, Albert DA (2010). Hyperuricemia and coronary heart disease: a systematic review and meta-analysis. Arthritis Care Res (Hoboken).

[20] Iseki K, Ikemiya Y, Inoue T, Iseki C, Kinjo K, Takishita S (2004). Significance of hyperuricemia as a risk factor for developing ESRD in a screened cohort. Am J Kidney Dis.

[21] Kansui Y, Matsumura K, Morinaga Y, Inoue M, Kiyohara K, Ohta Y (2018). Impact of serum uric acid on incident hypertension in a worksite population of Japanese men. J Hypertens.

[22] Chen Q, Yin YJ, Chen WY, Wu JN, Huang X (2018). Assessment of the association between serum uric acid levels and the incidence of hypertension in nonmetabolic syndrome subjects: a prospective observational study. Med (Baltim).

[23] Sung KC, Byrne CD, Ryu S, Lee JY, Lee SH, Kim JY (2017). Baseline and change in uric acid concentration over time are associated with incident hypertension in large Korean cohort. Am J Hypertens.

[24] Cui LF, Shi HJ, Wu SL, Shu R, Liu N, Wang GY (2017). Association of serum uric acid and risk of hypertension in adults: a prospective study of Kailuan Corporation cohort. Clin Rheumatol.

[25] Yokoi Y, Kondo T, Okumura N, Shimokata K, Osugi S, Maeda K (2016). Serum uric acid as a predictor of future hypertension: Stratified analysis based on body mass index and age. Prev Med.

[26] Yang T, Chu CH, Bai CH, You SL, Chou YC, Hwang LC (2012). Uric acid concentration as a risk marker for blood pressure progression and incident hypertension: a Chinese cohort study. Metabolism.

[27] Lee JJ, Ahn J, Hwang J, Han SW, Lee KN, Kim JB (2015). Relationship between uric acid and blood pressure in different age groups. Clin Hypertens.

