# Supplementary material for: Validation of age- and sex-dependent association of uric acid and incident hypertension in rural areas
Source: Clin Hypertens. 2022 Sep 1;28:24. doi: 10.1186/s40885-022-00206-5 (PMC9434906; doi:10.1186/s40885-022-00206-5)
Supplement: Supplementary file 1 — Additional file 1: Supplementary Table 1. Comparison of baseline characteristics between urban-based and rural-based cohorts. [file 40885_2022_206_MOESM1_ESM.docx]

**Supplementary Table 1.** Comparison of baseline characteristics between urban-based and rural-based cohorts

| Variable | Rural-based cohort  (n = 4,592) | Urban-based cohort  (n = 29,088) |
| --- | --- | --- |
| Male sex | 1,733 (37.7) | 9,137 (31.4) |
| Age (yr) | 60.1 ± 9.5 | 52.5 ± 7.8 |
| Smoking status |  |  |
| Non-smoker | 2,401 (67.5) | 21,987 (75.9) |
| Ex-smoker | 578 (16.3) | 3,903 (13.5) |
| Current smoker | 576 (16.2) | 3,058 (10.6) |
| Drinking status |  |  |
| Non-drinker | 2,409 (52.5) | 15,175 (52.4) |
| Ex-drinker | 294 (6.4) | 1,028 (3.6) |
| Current drinker | 1,886 (41.1) | 12,739 (44.0) |
| Height (cm) | 157.6 ± 8.3 | 160.6 ± 7.8 |
| Weight (kg) | 59.7 ± 9.7 | 60.6 ± 9.5 |
| Body mass index (kg/m^2^) | 24.0 ± 3.0 | 23.4 ± 2.7 |
| Systolic blood pressure (mmHg) | 116.5 ± 11.6 | 117.5 ± 11.4 |
| Diastolic blood pressure (mmHg) | 74.6 ± 7.9 | 72.8 ± 7.8 |
| Fasting blood glucose (mg/dL) | 98.9 ± 23.5 | 93.0 ± 17.5 |
| eGFR (mL/min/1.73 m^2^) | 76.4 ± 11.3 | 93.3 ± 13.0 |
| Total cholesterol (mg/dL) | 197.4 ± 35.7 | 197.6 ± 35.1 |
| High density lipoprotein (mg/dL) | 45.3 ± 10.8 | 54.2 ± 13.0 |
| Low density lipoprotein (mg/dL) | 124.0 ± 32.9 | 119.8 ± 32.0 |
| Triglyceride (mg/dL) | 140.5 ± 87.2 | 118.3 ± 80.1 |
| Diabetes mellitus | 438 (9.5) | 1,876 (6.5) |
| Dyslipidemia | 2,988 (65.1) | 13,071 (44.9) |
| Serum uric acid (mg/dL) | 4.8 ± 1.3 | 4.5 ± 1.2 |
| Hyperuricemia | 418 (9.1) | 1,465 (5.0) |

Data are presented as number (%) or mean ± standard deviation.

eGFR, estimated glomerular filtration rate.
